# Reusable fiberglass and polyester combined total contact cast system for treatment of plantar diabetic foot ulcers: efficacy and cost-effectiveness

**DOI:** 10.3389/fendo.2025.1674774

**Published:** 2025-09-22

**Authors:** Alper Erkin, Hande Cengiz Açıl, Ayşe Çelik Yılmaz, Taner Demirci, Thomas Eberleın

**Affiliations:** ^1^ Department of Wound Care, Sakarya University Training and Research Hospital, Sakarya, Türkiye; ^2^ Department of Surgical Nursing, Sakarya University, Faculty of Health Sciences, Sakarya, Türkiye; ^3^ Department of Endocrinology and Metabolism, Sakarya University Faculty of Medicine, Sakarya, Türkiye; ^4^ IHM International Institute for technology-based Education in Health, Management and Social Services, Wels, Austria

**Keywords:** diabetic foot, foot ulcer, off-loading, plantar pressure, casting devices

## Abstract

**Background:**

The basic principles of TCC are to achieve complete contact of the cast with the entire plantar surface of the foot and distribute the pressure at the sole. This method also reduces the shear forces generated at the wound edges and increases the healing potential of the wound.

**Purpose:**

This study aimed to evaluate the effectiveness of a reusable fiberglass and polyester combined total contact cast (TCC) system in the treatment of plantar diabetic foot ulcers.

**Study Design:**

A retrospective review was conducted on 70 patients treated with the reusable TCC system between January 2020 and September 2022.

**Methods:**

The hospital’s electronic medical record system was searched for cases using CPT code 29445 (application of a rigid total contact cast, half leg, adult). Patients included had persistent plantar ulcers at pressure points unresponsive to standard care and were treated with the TCC system until granulation tissue developed. Cases involving deep tissue infections or osteomyelitis were managed according to international diabetic foot guidelines.

**Results:**

Out of 70 patients, 53 (75.7%) achieved complete wound closure. Seventeen patients showed no healing; among these, 9 underwent minor amputations and 1 required a major amputation. No significant difference in healing time was found based on ulcer location (forefoot, midfoot, hindfoot) (p=0.503).

**Conclusion:**

The reusable fiberglass and polyester TCC system is a practical and cost-effective option for diabetic foot ulcers, offering outcomes similar to traditional TCCs. Continued use is recommended, with future research focusing on improving patient adherence and optimizing comfort in hybrid designs.

## Introduction

Diabetic foot ulcers (DFU) stand out as one of the most common complications of diabetes worldwide, and it is expected that approximately 550 million people will be diagnosed with diabetes by 2030. It is predicted that 12% to 25% of these people will develop DFU (1, 2). DFUs are a leading cause of diabetes-related hospitalizations and lower extremity amputations (3). Mechanical trauma plays a crucial role in the formation of DFU. High pressure, which reaches peak levels in a minimal plantar surface area, may increase over time and become the leading carrier of mechanical trauma (4, 5). The balanced distribution of this plantar pressure, i.e., partial removal from the DFU borders, aims to accelerate wound healing. Therefore, pressure relief at sole is one of the essential determinants in treatment of plantar DFU (6).

The basic principles of TCC are to achieve complete contact of the cast with the entire plantar surface of the foot and distribute the pressure at the sole. This method also reduces the shear forces generated at the wound edges and increases the healing potential of the wound (7). Shear forces are known to be a factor that impedes wound healing, especially in neuropathic foot ulcers. Therefore, the positive effect of TCC on healing is due to its capacity to promote wound healing by improving plantar pressure distribution and controlling shear forces. Different designs have been developed to apply TCC and provide plantar offloading (8).

Total contact cast (TCC) is considered as a well-formed, hand-made method that protects the plantar surface by remaining in contact with all surfaces of foot and cruris, thus prevents further pressure exposure (9). The effectiveness of TCC in treating neuropathic foot ulcerations is due to both reducing pressure on the plantar surface of the foot and providing movement restriction when the cast cannot be removed (7, 10). Studies have shown that modifying the cast results in a 70% reduction in peak pressure in ulcerated regions and a 69% improvement in pressure-time integrals. In addition, increased patient satisfaction and shorter wound healing times have been reported with modified TCC (11, 12).

Total Contact Cast (TCC) is widely recognized as the gold standard for offloading plantar pressure in patients with diabetic foot ulcers (DFUs). TCC redistributes plantar pressure, reduces focal tissue stress, and promotes ulcer healing. Healing rates remain suboptimal, and DFUs impose a substantial economic burden, particularly in low-resource settings where access to specialized care and advanced wound management is limited (13). Reusable fiberglass and polyester hybrid TCC systems offer several advantages over traditional single-use casts, including improved cost-effectiveness, enhanced patient adherence, and reduced medical waste, contributing to environmental sustainability. These benefits make reusable TCC a practical alternative for widespread clinical adoption.

The fiberglass and polyester combined TCC system is a convenient and cost-effective system to control disribution of plantar pressures and allow consecutive dressing changes with its long term usability. This study aimed to evaluate the effectiveness of a reusable fiberglass and polyester TCC system in treating plantar DFUs, with a focus on its impact on wound healing, cost-effectiveness, and patient compliance. The choice of hybrid materials was intended to enhance durability and reusability, thereby promoting adherence and reducing overall healthcare costs.

## Methogology

This was a single-center, IRB-approved retrospective assessment of all patients who had an ulcer treated with a reusable TCC at our institution between January 2020 and September 2022. The electronic medical record was searched for all cases in which the CPT code 29445, application of a rigid total contact cast, half leg, adult, was used. All patients were treated till the formation of granulation tissue in the wound bed. Deep tissue infections and osteomyelitis were treated under the guidance of an international working group on diabetic foot. Patients who had persistent ulcers at the pressure points that could not be healed despite appropriate wound care practices and were therefore treated with TCC were included in the study. Age, gender, body mass index, duration of diabetes, glomerular filtration ratio (GFR), HbA1C, low-density lipoprotein (LDL), peripheral arterial disease, peripheral sensory neuropathy, plantar ulcer localization, minor amputation, major amputation, mortality data were all collected from patients.

Among the 70 patients treated with the reusable total contact cast (TCC), 5 had peripheral arterial disease (PAD); 4 of them achieved complete healing, while 1 did not, suggesting that PAD may have a limited impact on wound healing in this cohort. PAD severity was not routinely assessed using ankle-brachial index or pedal acceleration time, which is a study limitation. Patients who did not heal or required amputation often had persistent infection, osteomyelitis, or PAD. Total contact casts were applied by a wound care team consisting of a specialist physician and trained nurses, ensuring standard application and monitoring. These details emphasize the impact of comorbidities and personnel expertise on healing outcomes and support the effectiveness and safety of the reusable fiberglass and polyester TCC system.

The study included patients with persistent plantar ulcers at pressure points that did not respond to standard wound care practices. Patients with deep tissue infections or osteomyelitis were managed according to international diabetic foot guidelines before TCC application. Those with severe peripheral arterial disease, for whom TCC was contraindicated, were excluded. All patients received a standardized wound care protocol prior to TCC initiation, ensuring consistent pre-treatment management and enabling accurate evaluation of the reusable fiberglass and polyester TCC system’s effectiveness.

Wounds were assessed using the Wagner classification system. Standardized dressing materials were used for all patients, with dressing changes performed according to wound exudate and clinical guidelines. Granulation tissue development was determined clinically by the presence of healthy, beefy-red tissue covering the wound bed, and complete wound closure was defined as full epithelialization without drainage. Patient adherence to the TCC protocol was monitored at follow-up visits, during which patients were instructed not to remove the device. Follow-up intervals were scheduled every 1–2 weeks, depending on wound severity. The TCC application procedure followed standardized steps; step-by-step figures have been included to enhance reproducibility for other clinicians.

### Application procedure of reusable fiberglass and polyester combined total contact cast system

**Figure 1:**
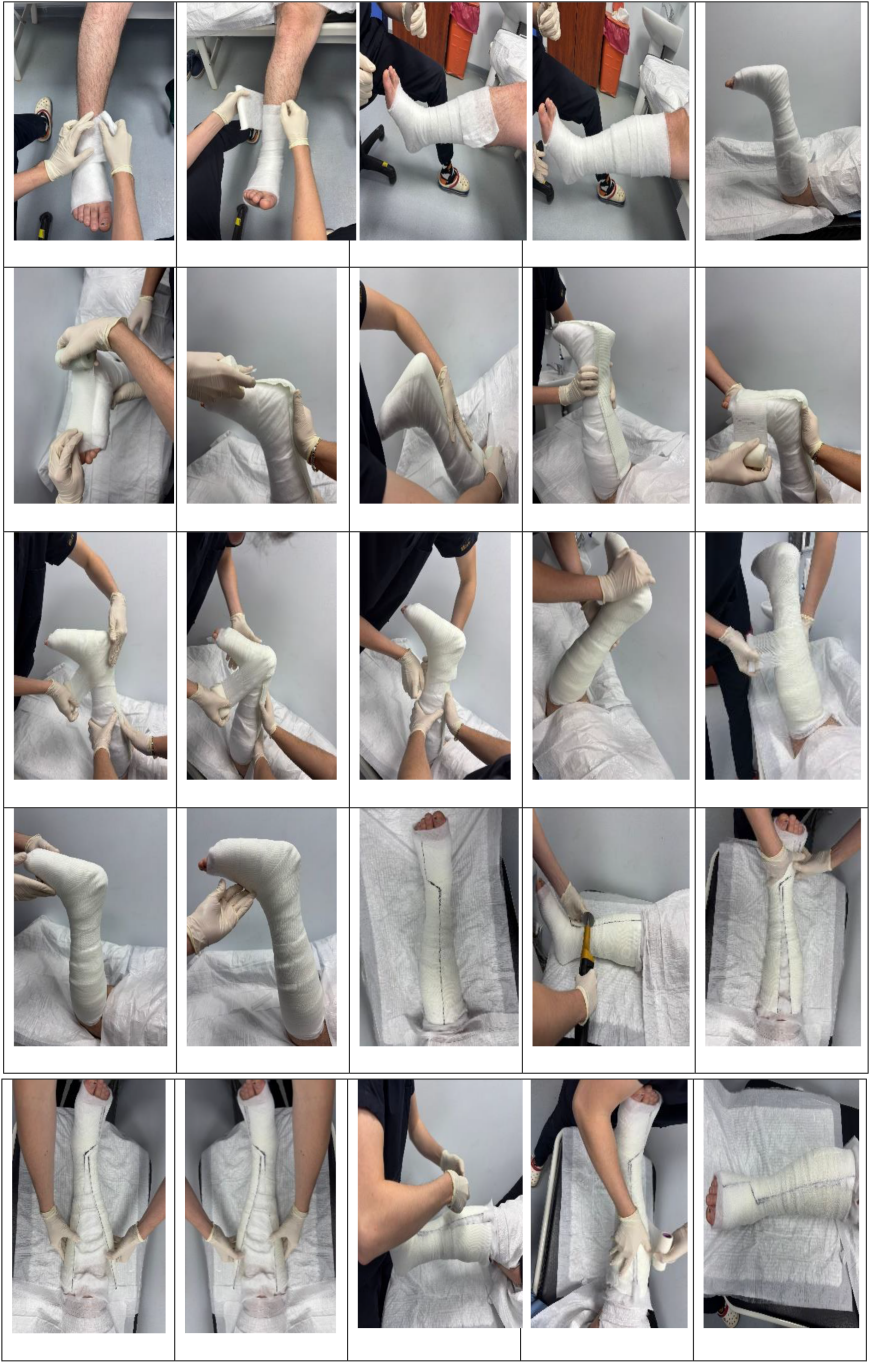
Reusable fiberglass and polyester combined total contact cast system.

Before application; wound bed was cleansed and debrided, hypertrophic callus belonging to the wound bed was resected and appropriate dressing was applied in accordance with TIMERS principles. A TCC was designed as a semi-rigid toe-to-knee cast without movement in the ankle joint. Rigid fiberglass synthetic cast was used in combination with soft polyester cast. A fiberglass synthetic cast was placed longitudinally from the toes up to tuberositas tibia level with total contact to the sole and posterior surface of cruris. Then, two soft polyester synthetic casts were wrapped circularly around the foot and cruris (**Figure 1**).

### Statistical analysis

Descriptive data are presented as percentages, or in the case of continuous variables, as medians with interquartile ranges [IQR]. Continuous variables were reported as mean ± standard deviation, and categorical variables as frequencies and percentages. Missing data were handled using a complete case analysis approach. To control for potential confounding factors such as neuropathy, peripheral arterial disease (PAD), and glycemic control, multivariate analyses were performed. These steps ensured a robust evaluation of the effectiveness of the reusable fiberglass and polyester TCC system.

## Results

The study included 70 patients who had previously been applied the fiberglass and polyester combined TCC system to treat unhealed diabetic foot ulcers despite appropriate wound treatment at our institute. Forty-seven patients (67.1%) were male. Patients in the study group had a mean age of 57.3 ± 12.8 years. The average duration of diabetes mellitus was 20.0 ± 8.3 years. The patients’ GFR rates were 83.7 ± 32.5, their HbA1c levels were % 8.4 ± 1.8 and their LDL was 118.5 ± 33.6 (**Table 1**).

**Table 1:** Demographic data.

Variables	Results (n=70)
Age, year	57.3 ± 12.8
Gender, Male, n(%)	47(67.1)
BMI,kg/m^2^	29.4 ± 6.4
Duration of Diabetes, year	20.00 ± 8.3
GFR	83.7 ± 32.5
HbA1C, %	8.4 ± 1.8
LDL,mg/dL	118.5 ± 33.6

BMI, body mass index; GFR, Glomerular filtration ratio.

Thirty-eight (54.3%) patients had underlying peripheral neuropathy, and 5 (7.1%) had peripheral arterial disease. Most (n=38) of DFU were located in the forefoot, with 11 in the midfoot and 10 in the hindfoot region, respectively. Of the 70 plantar ulcers, 38 (54.3%) were in the forefoot, eight (11.4%) were in the midfoot, and 24 (34.3%) were in the hindfoot. While the number of patients who underwent minor amputation was 9 (12.9%), one patient (1.4%) underwent major amputation (**Table 2**).

**Table 2:** Chronic complications and results.

Variables	Results (n=70)
Peripheral arterial disease,exist, n (%)	5(7.1)
Peripheral neuropathy,exist, n (%)	38(54.3)
Plantar ulcer localization, n (%)	
Forefoot	38(54.3)
Midfoot	8(11.4)
Hindfoot	24(34.3)
Minor amputation, n (%)	9 (12.9)
Major amputation, n (%)	1(1.4)
Mortality, n (%)	3(4.3)

**Figure 2:**
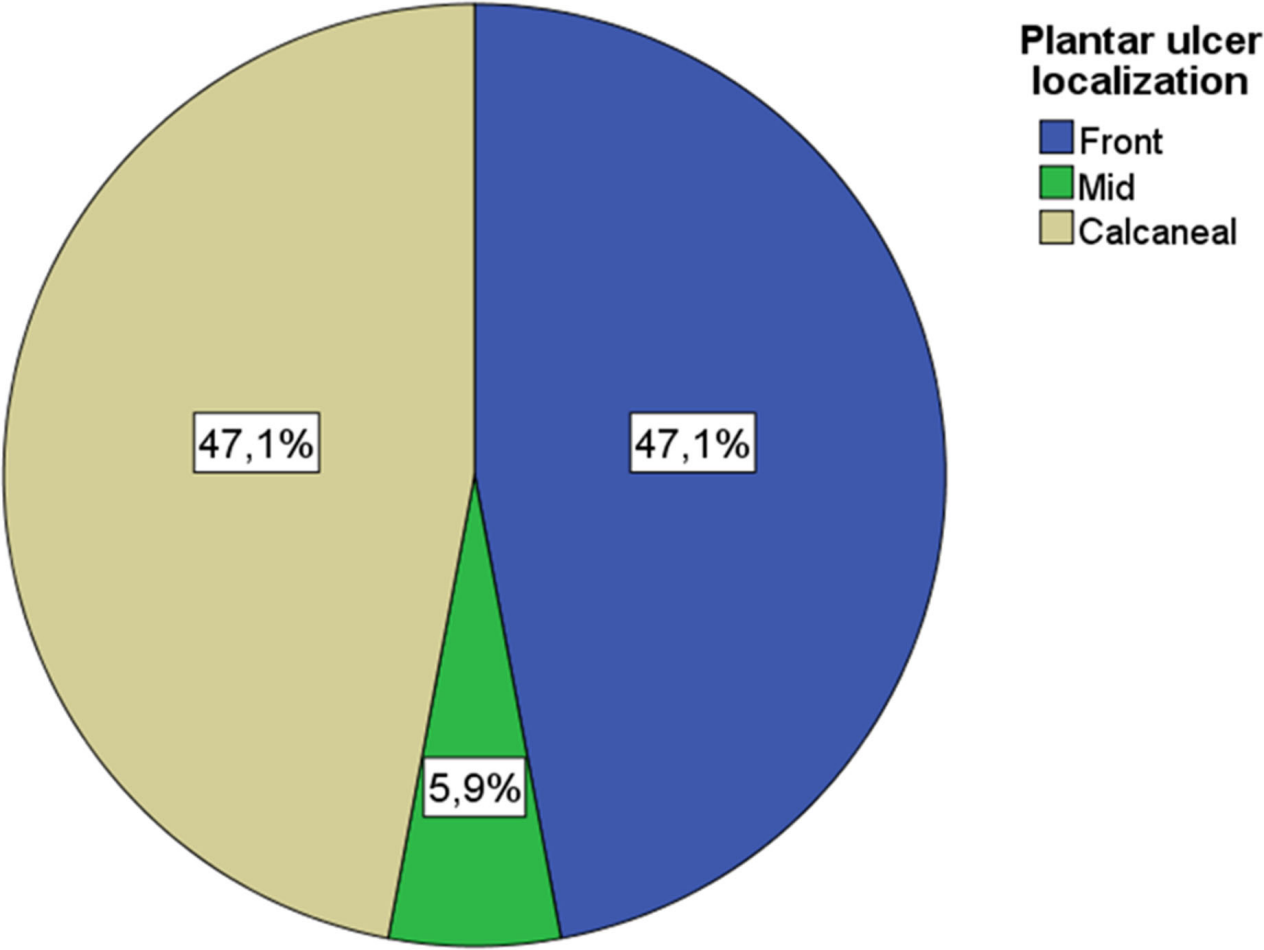
Plantar ulcer localization.

Among the patients treated with a removable TCC system, 17 wounds did not reach complete closure during the follow-up period. Eight of the non-closure wounds (47.1) were located in the forefoot, one (5.9%) in the midfoot, and eight (47.1%) in the hindfoot, respectively (**Figure 2**).

**Figure 3:**
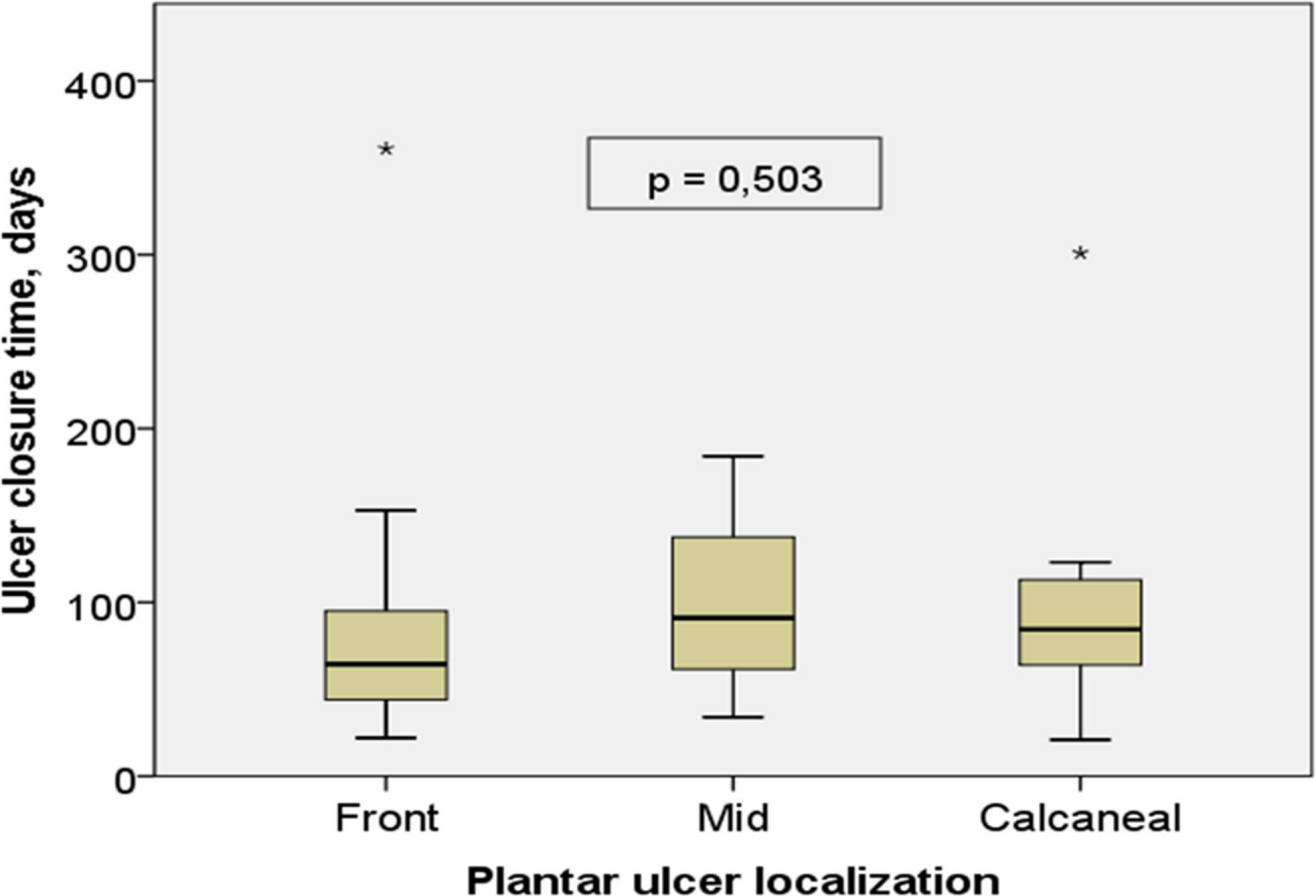
Plantar ulcer closure time and ulcer localization. The meaning of the * symbol: The box plot shows extreme data in the chart.

During the follow-up period, there was no statistically significant difference between the healing times of plantar regions of the foot (p=0.503). The median wound closure time for forefoot ulcers was (IQR) 64.5 (41-100.75), the median wound closure time for midfoot ulcers was (IQR) 91.00 (47-182) and the median wound closure time for hindfoot ulcers was (IQR) 84,5 (63.5-115.5), consecutively (**Figure 3**).

Complete wound closure was achieved in 53 of 70 patients (75.7%; 95% CI: 64.5–84.2), while no healing occurred in 17 patients. Ulcer characteristics, including size, depth, and chronicity, were recorded, as these factors influence healing outcomes. Median healing times were calculated, and Kaplan–Meier survival analysis was performed to compare healing times across different ulcer locations (forefoot, midfoot, hindfoot), although no statistically significant differences were observed (p=0.503). While formal cost analysis was not conducted, the discussion emphasizes the practical cost-effectiveness of the reusable fiberglass and polyester TCC system by reducing the need for repeated cast application and lowering material waste.

## Discussion

In this study, among the patients treated with the removable TCC system, complete closure was not achieved during the follow-up period of 17 wounds, while complete healing was observed in 53 patients. While the number of patients who underwent minor amputation was 9, one patient underwent major amputation. In the literature, Fife et al. (2014) reported lower infection and amputation rates in patients who underwent TCC in a study with a large sample group (11784) (14). In another study using a single TCC system, 113 out of 132 ulcerations (85.6%) healed, 6 (5.5%) did not heal, and 13 resulted in amputation (9.8%) (1). These results suggest that the total contact cast system consisting of reusable fiberglass and polyester has similar outcomes to removable cast walkers and single-use prefabricated rigid TCC systems.

When looking at the studies examining healing times in the literature; In one study, the healing time of DFU with an off-loading device varied from 1 week to 156 weeks with a median duration of 17.5 (95% confidence interval = 15-33) weeks (122.5 days) (15). In a comparative study conducted by Sahu et al. (2018) between conventional dressing and TCC for DFUs, better results were observed in the TCC group, with a mean healing time of 48 ± 7 days compared to the traditional group of dressing with a mean healing time of 58 ± 9 days (5). A similar study concluded that ulcer healing rates on the forefoot were better than on other foot areas (16). Another study shows that using the offloading technique, the average length of the wound healing process is a minimum of 24 days and a maximum of 52 days. Still, there is no difference in the size of the wound-healing process between the three wound areas (phalanges, metatarsals, and calcaneus) (17). Similarly, in this study, the median wound closure time (IQR) for forefoot ulcers was 64.5 (41-100.75), the median wound closure time (IQR) for midfoot ulcers was 91.00 (47-182) and the median wound closure time (IQR) for hindfoot ulcers was 84.5 (63.5-115.5), while no statistically significant difference was found between the healing times of the plantar regions of the foot during the follow-up period.

When the literature was examined in terms of the materials used; in the study of Pirozzi et al.’s study (18), TCC was developed from two different materials, polyurethane foam and fiberglass, and it was found that both materials did not make a difference in patient outcomes (18). A study using non-removable fiberglass TCC and a total contact soft cast (TCS) on diabetic foot wounds found similar healing rates (19). Similar to our study, Ting et al. (2024) reported that the moldable fiberglass support plate device was effective in the drainage of plantar foot ulcers and could potentially provide better drainage when used in a removable above-knee or above-ankle device compared to using these devices alone (20). In this study, the Reusable Fiberglass and Polyester Combined Full Contact Cast System Application was performed and it was found to have similar healing rates and could be used as an alternative to conventional TCC or any other offloading device in the treatment of plantar diabetic foot ulcerations.

While formal cost analysis was not performed, the reusable fiberglass and polyester TCC system provides practical cost-effectiveness by reducing the need for repeated cast applications and minimizing material waste. Patient comfort and mobility were generally maintained, with minor adjustments applied as needed to improve tolerance. Reusing casts introduces practical challenges, including sterilization and durability, which were addressed through standardized cleaning protocols and routine material inspections. Among the 24.3% of patients who did not achieve healing, contributing factors included severe peripheral arterial disease, persistent infection, osteomyelitis, and ulcer chronicity. Strategies to improve outcomes may involve enhanced infection control, adjunctive therapies, and careful patient selection. Strict sterilization procedures mitigated potential infection risks associated with reusability. The environmental and economic benefits of the reusable TCC, including reduced medical waste and lower material costs compared to traditional single-use casts, further support its sustainability and practical value in clinical settings.

## Conclusion

This study demonstrates that a reusable fiberglass and polyester combination total contact cast (TCC) system may be an effective alternative in the treatment of plantar diabetic foot ulcers. In addition, as reported in previous studies, the use of TCC reduces infection and amputation rates, and provides superior results compared to traditional dressings or removable devices, especially in cases where compliance is high. The reusable fiberglass and polyester TCC system effectively promotes healing of plantar diabetic foot ulcers while offering practical advantages in cost-effectiveness, patient adherence, and reduced medical waste. Nevertheless, these findings should be interpreted with caution and require validation through larger, prospective, multicenter studies.

## Future directions

Future research may include randomized controlled trials comparing reusable TCC with conventional TCC and removable walkers, incorporation of patient-reported outcome measures to evaluate comfort and quality of life, and material science studies addressing durability, cleaning protocols, and long-term reusability of the hybrid casts.

## Limitations

This study has several limitations that should be considered when interpreting the findings. First, it was conducted at a single center, which may introduce selection bias and limit generalizability. Second, the retrospective design restricts causal inference. Third, baseline characteristics did not include detailed ulcer grading, size, or infection status, which are important predictors of healing outcomes. Finally, no formal cost or quality-of-life assessments were performed, although practical cost-effectiveness and patient adherence were discussed. Future studies should address these limitations with prospective, multicenter designs and comprehensive outcome measures.

## Data Availability

The original contributions presented in the study are included in the article/supplementary material. Further inquiries can be directed to the corresponding author.
